# Provision of Paid Web-Based Medical Consultation in China: Cross-Sectional Analysis of Data From a Medical Consultation Website

**DOI:** 10.2196/12126

**Published:** 2019-06-03

**Authors:** Yumei Li, Xiangbin Yan, Xiaolong Song

**Affiliations:** 1 Harbin Institute of Technology Harbin China; 2 University of Science and Technology Beijing Beijing China; 3 School of Management Science and Engineering, Dongbei University of Finance and Economics Dalian China

**Keywords:** e-consultation, medical service, fee, China

## Abstract

**Background:**

Web-based medical consultation, which has been adopted by patients in many countries, requires a large number of doctors to provide services. However, no study has provided an overall picture of the doctors who provide online services.

**Objective:**

This study sought to examine doctors’ participation in medical consultation practice in an online consultation platform. This paper aimed to address the following questions: (1) which doctors provide Web-based consultation services, (2) how many patients do the doctors get online, and (3) what price do they charge. We further explored the development of market segments in various departments and various provinces.

**Methods:**

This study explored the dataset including all doctors providing consultation services in their spare time on a Chinese Web-based consultation platform. We also brought in statistics for doctors providing offline consultations in China. We made use of Bonferroni multiple comparison procedures and z test to compare doctors in each group.

**Results:**

There are 88,308 doctors providing Web-based consultation in their spare time on Haodf, accounting for 5.25% (88,308/1,680,062) of all doctors in China as of September 23, 2017. Of these online doctors, 49.9% (44,066/88,308) are high-quality doctors having a title of chief physician or associate chief physician, and 84.8% (74,899/88,308) come from the top, level 3, hospitals. Online doctors had an average workload of 0.38 patients per doctor per day, with an online and offline ratio of 1:14. The average price of online consultation is ¥32.3. The online ratios for the cancer, internal medicine, ophthalmology-otorhinolaryngology, psychiatry, gynecology-obstetrics-pediatrics, dermatology, surgery, and traditional Chinese medicine departments are 16.1% (2,983/18,481), 4.4% (16,231/372,974), 6.3% (8,389/132,725), 9.5% (1,600/16,801), 5.8% (12,955/225,128), 18.0% (3,334/18,481), 10.8% (24,030/223,448), and 3.8% (8,393/22,3448), respectively. Most provinces located in eastern China have more than 4000 doctors online. The online workloads for doctors from most provinces range from 0.3 to 0.4 patients per doctor per day. In most provinces, doctors’ charges range from ¥20 to ¥30.

**Conclusions:**

Quality doctors are more likely to provide Web-based consultation services, get more patients, and charge higher service fees in an online consultation platform. Policies and promotions could attract more doctors to provide Web-based consultation. The online submarket for the departments of dermatology, psychiatry, and gynecology-obstetrics-pediatrics developed better than other departments such as internal medicine and traditional Chinese medicine. The result could be a reference for policy making to improve the medical system both online and offline. As all the data used for analysis were from a single website, the data might be biased and might not be a representative national sample of China.

## Introduction

### Background

Web-based consultation services were first provided to the public by University Hospital of Zurich in 1999 [1]. They have been adopted in many countries to provide better service for patients. An Web-based consultation can serve as an effective complement to traditional health care [2] and conventional physician-patient relationships [3]. Offering Web-based medical services also could make economic sense [4]. Some researchers suggest that 25% to 70% of all patients seeking care do not need a face-to-face appointment with a doctor [5], and there is a great demand for online consultation services.

However, the doctor has been found to be less likely to accept Web-based consultation services than the patient [6]. Consultations, as essentially private information goods, are produced only for a specific consumer [4], and Web-based consultation platforms require a number of doctors to keep patients to attain adequate services. Many of the Web-based consultation platforms are not yet mature and suffer from online doctor scarcity [7]. Furthermore, the Web-based service market was more concentrated on highly skilled doctors’ service than the offline service market [8], which makes things worse. Attention needs to be focused on attracting doctors to promote development of Web-based consultation services. Guo et al found that doctors could gain economic and social returns from providing Web-based medical services [9]. Akcura and Ozdemir took an economic perspective to explain doctors adopting Web-based consultation services [4,7,10]. Ozdemir explored doctors’ optimal channel strategies via online and offline consultation to get maximum economic profit [4]. Akcura et al examined the conditions under which a quality traditional expert will augment its offline channel by offering an online version of its services [10]. Akcura and Ozdemir investigate when and how expert service providers should offer their services online and what price strategy they should adopt [11]. Akcura and Ozdemir’s works are based on game theoretic models and a series of assumptions. However, there is a shortage of description and understanding about doctors who provide Web-based consultation services [7]. In conjunction with the expectation of high-quality internet information, more and more patients are willing to pay for high-quality information provided by specialists online [12]. China is becoming the fastest growing market for Web-based doctor consultation [13]. The market characteristics are also needed to be understood for future development of Web-based consultation services.

### Objectives

We tried to fill this gap by investigating doctors’ consultation services on Haodf —a Web-based consultation platform in China. The doctors, as the sellers in the Web-based consultation service market, are the key participators we need to focus on. To know more about the doctors, we seek to answer these questions in this paper: (1) which doctors provide Web-based consultation services, (2) how many patients do they serve, and (3) how much do they charge for the Web-based service? We discuss the development of Web-based consultation across departments and provinces. We considered doctors’ technical levels, hospitals, departments, and locations to answer the questions. We also compared the condition of doctors’ online service with the average level of offline service to understand the online data more exactly.

## Methods

### Data

As mentioned in the previous section, we obtained the data of doctors who provided Web-based consultation service from Haodf —a Web-based medical service platform. The platform is the *Yelp* for doctor information in China, which lists information of doctors from all over the country and was first built in 2006. Haodf promoted the online review system and the online doctor-patient communication system. Currently, it covers more than half a million doctors for patients to review, according to their own report. Nearly 200,000 doctors have registered and connected with patients through it. Doctors provide Web-based consultation on the website through phones, messages, and graphs in their spare time at a price set by themselves. The rich information about the doctor makes Haodf a perfect platform for doctors to improve reputation. The adoption rate of Haodf as a Web-based consultation platform by doctors is higher than other Web-based consultation platforms [14].

The data of more than 170,000 doctors registered on the platform were collected on March 23, 2017, and September 23, 2017 (we just included doctors in Mainland China, excluding doctors from Hong Kong, Macao, and Taiwan). We removed the data of doctors (1) who did not provide Web-based consultation service when we collected the data; (2) who had not been marked as chief physician, associate chief physician, attending physician, or resident physician; and (3) whose records had missing or abnormal values. We finally got 88,308 doctors who provided Web-based consultation services on the website. The data included a doctor’s title, hospital, department, location, services provided, fee for the consultation service, and number of patients served in the past half year. These variables are described in Table 1.

**Table 1:** Description of the variables.

Variables	Description
Title	Doctor’s technical title, values could be chief physician, associate chief physician, attending physician, and resident physician (from senior to junior levels). The title could indicate the doctor’s work experience and technical level [15].
Hospital	The hospitals in China are divided into 3 levels by the government according to hospital functions, facilities, and technical strength. Hospitals where the doctor works, values could be level 1 hospital, level 2 hospital, or level 3 hospital (level 3 is the best).
Department	Department, values could be cancer, internal medicine, ophthalmology and otorhinolaryngology, psychiatric, gynecology-obstetrics-pediatrics, dermatology, surgery, traditional Chinese medicine, and others.
Location	The 31 provinces and municipalities in Mainland China, excluding Hong Kong, Macao, and Taiwan.
Online ratio	Number of online doctors/numbers of offline doctors.
Workload	Number of patients a doctor serves per day on Haodf. (We took a doctor’s total patient number on March 23, 2017, and September 23, 2017, then, we subtract the previous number from the last one to get the total number of patients in the half year. Finally, we get doctor’s total patient number divided by 183.)
Fee	Consulting fee.

### Method

Swanson pointed out in 1971 that “*Thinking without comparison is unthinkable. And, in the absence of comparison, so is all scientific thought and scientific research*.” To understand the status of Web-based consultation services, we described the doctors’ providing Web-based service, patients number, and service price and showed the difference of that across doctors with different technical levels, hospitals, departments, and locations. We also compared consultation services of online doctors and offline doctors. We got the corresponding statistics of doctors providing offline service from the *China Health and Family Planning Statistics Yearbook 2017* [16]. The data include the number of doctors in hospitals; departments and provinces at the end of 2016; as well as the number of patients in hospitals, departments, and provinces in 2016.

To answer “who provides Web-based consultation service,” we listed the number of online and offline doctors, and then calculated the online ratio of doctors with different titles, hospital levels, departments, and geographical locations. To answer “how many patients do the Web-based doctors serve,” we described the workload distribution on Haodf among doctors with different titles, hospitals, departments, and locations. Then, we made use of the Bonferroni multiple comparison procedures (BMCP) [17] to compare the patient’s number of doctors across the subgroups. To exclude the impact of offline doctor-patient distribution among subgroups, we further considered the offline patient number per doctor. With the z test, we compared the online patients’ number with adjusted online patients’ number per doctor (offline doctor number × average of online patients’ number per doctor/average of online patients’ number per doctor). As there was no exact offline patient data from March 2017 to September 2017, we adopted the distribution data of patients in 2016.

To answer “what’s the price for the services,” we described the price distribution among doctors with various titles, different hospitals, different departments, and different geographical positions. Then, we made use of BMCP to compare the price of the service across the subgroups.

## Results

### Who Provides Web-Based Consultation Service?

**Figure 1:**
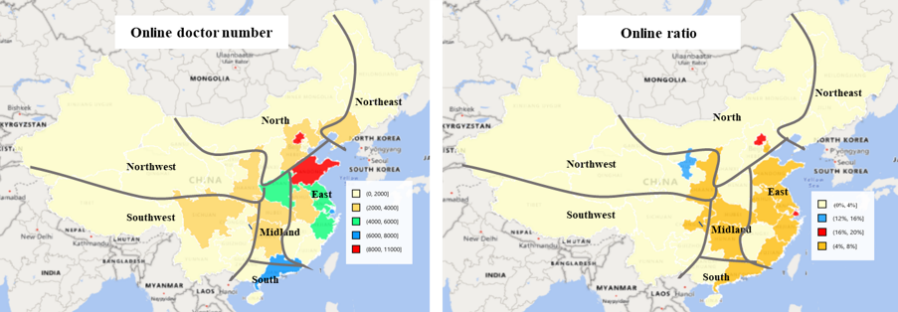
The doctors’ distribution in Mainland China.

We described the characteristic of doctors who provide Web-based consultation and then compare online and offline doctors based on technical level, hospital, department, and location. The results are listed in Table 2 and Figure 1. There are 1,680,062 doctors in China according to the *China Health and Family Planning Statistical Yearbook 2017* [16]. Of these, 5.3% provided Web-based consultation on Haodf as of September 23, 2017.

Referring to doctors' experience and skill level, 15.1% of chief physicians provide Web-based consultation, whereas the online ratio decreases as doctors’ experience decreases. The percentages of associate chief physicians, attending physicians, and resident physicians providing Web-based consultations are 8.4%, 5.3% and 2.2%, respectively. Chief physicians and associate chief physicians, who can be called experts, account for nearly 50% of all online doctors, whereas they only account for 25.5% of offline doctors. In the platform, attending physicians get the highest proportion (30,802/88,308, 34.9%) and resident physicians get the lowest proportion (13,440/88,308, 15.2%). In the offline environment, resident physicians get the highest proportion (601,462/1,680,062, 35.8%) and chief physicians get the lowest proportion (119,284/1,680,062, 7.1%).

In addition, 9.4% of level 3 hospital doctors provide Web-based consultations, which account for 84.8% of the total number of doctors who provide Web-based consultation. 1.8% of level 2 hospital doctors provide Web-based consultations, which account for 13.0% of the total number of doctors who provide Web-based consultation. However, doctors from level 1 hospitals and no-level hospitals account for just 2.2% of all online doctors.

**Table 2:** Doctor distribution in the online platform and offline hospitals.

Doctor numbers		Online, n (%)	Offline, n (%)	Online ratio (%)
**Total**				
	All doctors who provide consultation services	88,308 (100.0)	1,680,062 (100.0)	5.3
**Title**				
	Chief physician	18,030 (20.4)	119,284 (7.1)	15.1
	Associate chief physician	26,036 (29.5)	309,131 (18.4)	8.4
	Attending physician	30,802 (34.9)	581,301 (34.6)	5.3
	Resident physician	13,440 (15.2)	601,462 (35.8)	2.2
	Unknown	0(0)	68,883 (4.1)	0
**Hospital**				
	Level 3	74,899 (84.8)	795,043 (47.3)	9.4
	Level 2	11,454 (13.0)	643,167 (38.3)	1.8
	Level 1	718 (0.8)	115,114 (6.9)	0.6
	No level	1237 (1.4)	126,738 (7.5)	1.0
**Department**				
	Cancer	2983 (3.4)	18,481 (1.1)	16.1
	Internal medicine	16,231 (18.4)	372,974 (22.2)	4.4
	Ophthalmology-otorhinolaryngology	8389 (9.5)	132,725 (7.9)	6.3
	Psychiatry	1600 (1.8)	16,801 (1.0)	9.5
	Gynecology-obstetrics-pediatrics	12,955 (14.7)	225,128 (13.4)	5.8
	Dermatology	3334 (3.8)	18,481 (1.1)	18.0
	Surgery	24,030 (27.2)	223,448 (13.3)	10.8
	Traditional Chinese medicine	8393 (9.5)	223,448 (13.3)	3.8
	Others	10,393 (11.8)	448,577 (26.7)	2.3

Surgery (24,030) and internal medicine (16,231) had the largest number of online doctors. The online ratio of doctors in the dermatology (3,334/18,481, 18.0%), cancer (2,983/18,481, 16.1%), surgery (24,030/223,448, 10.8%), and psychiatry (1,600/16,801, 9.5%) departments providing Web-based consultation are higher than those in other departments. In these 4 departments, doctors in each department account for just 1% of all nationwide doctors, except for surgery (223,448/1,680,062, 13.3%). In the ophthalmology-otorhinolaryngology (8,389/132,725, 6.3%), gynecology-obstetrics-pediatrics (12,955/225,128, 5.8%), internal medicine (16,231/372,974, 4.4%), and traditional Chinese medicine (8,393/223,448, 3.8%) departments, doctors are less likely to provide Web-based services.

Figure 1 shows the number of online doctors and online ratio of doctors in Chinese provinces and cities. Online doctors mainly come from provinces and cities located in eastern China; in particular, the coastal provinces have more than 4000 online doctors. In most northern and western provinces of China, there are no more than 2000 online doctors. Most provinces and cities in east and midland China have more than 4% of online doctors, which is higher than other regions. Specifically, more than 16% of doctors are online in Beijing and Shanghai. Ningxia Autonomous Region, located in northwest China, also has more than 12% of doctors online.

### How Many Patients Does the Online Doctor Serve?

We describe the average patient number a doctor serves in 1 day as the workload for the doctor. Overall, 33.04% of doctors who provided Web-based consultation service got no patients in the past half year. The average online workload of doctors is higher than 75%, which means very few doctors got most of the patients.

We compared the workload of online doctors with that of offline doctors. The average workload of online doctors is 0.38 patients per doctor per day, whereas the average workload of offline doctors is 5.32 patients per doctor per day. The ratio of online doctors’ workload to offline doctors is 1:14.

**Figure 2:**
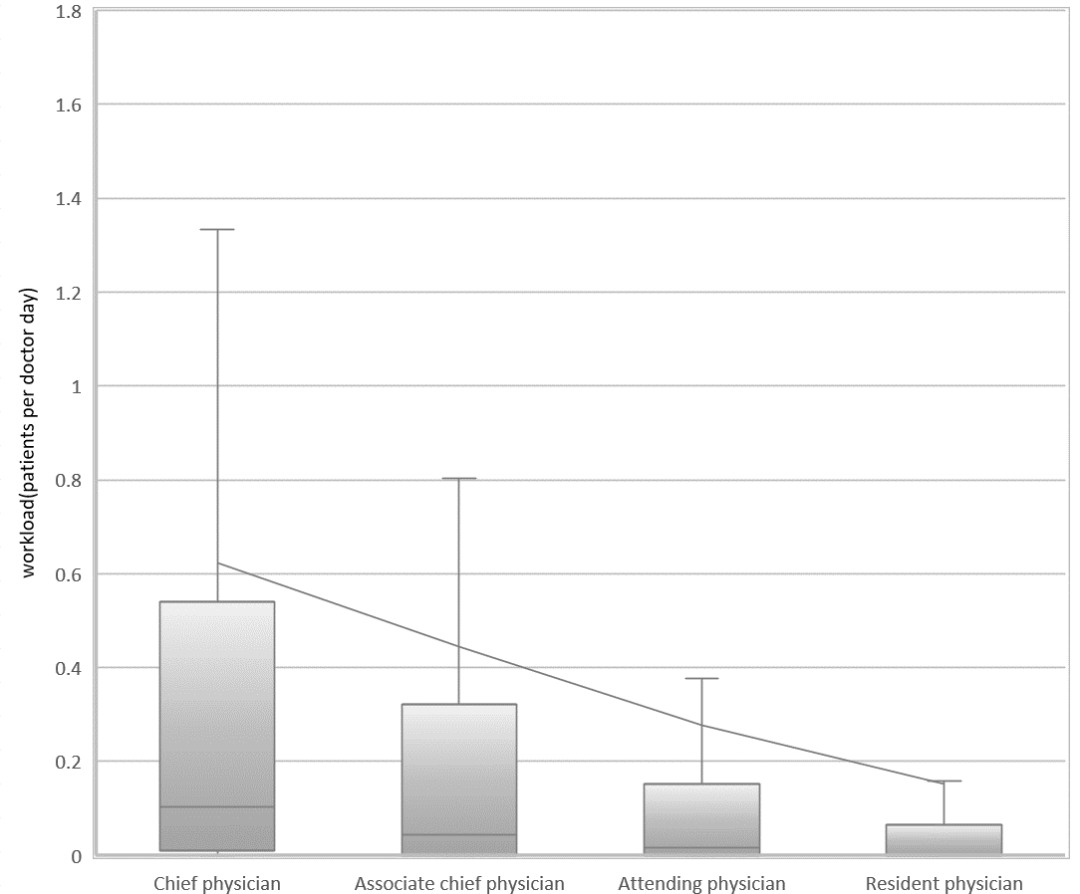
Distribution of online and offline patients per doctor across various titles.

Figure 2 shows the workload distribution of online doctors with different titles, the average workload of chief physicians (0.64), associate chief physicians (0.43), attending physicians (0.28), and resident physicians (0.15) decreased with the decline of doctors’ experience and skill level. With BMCP, the online workload for chief physicians is significantly higher than that of associate chief physicians (*P*<.01), the associate chief physicians are significantly higher than attending physicians (*P*<.01), the Attending Physicians are significantly higher than Resident Physicians (*P*<.01).

**Figure 3:**
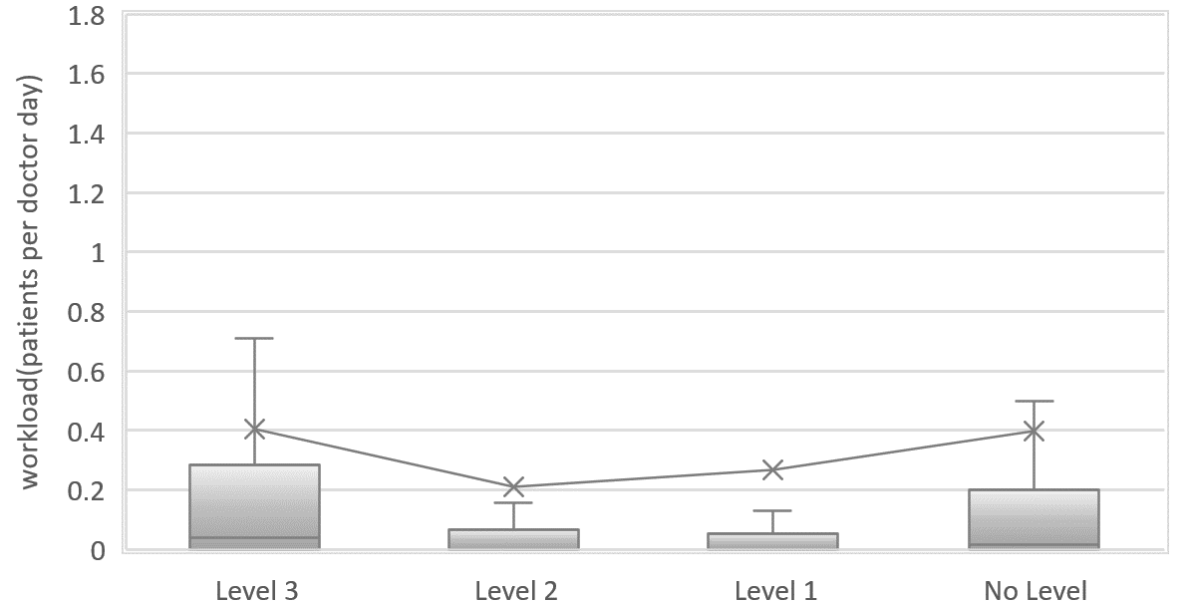
Distribution of online and offline patients per doctor across hospitals.

The workload distribution for online doctors from hospitals is shown in Figure 3. The workloads for doctors from level 3 hospitals are significantly higher than those from level 2 hospitals (*P*<.01) and level 1 hospitals (*P*<.01). Thus, high-level hospitals are more attractive to online patients.

**Figure 4:**
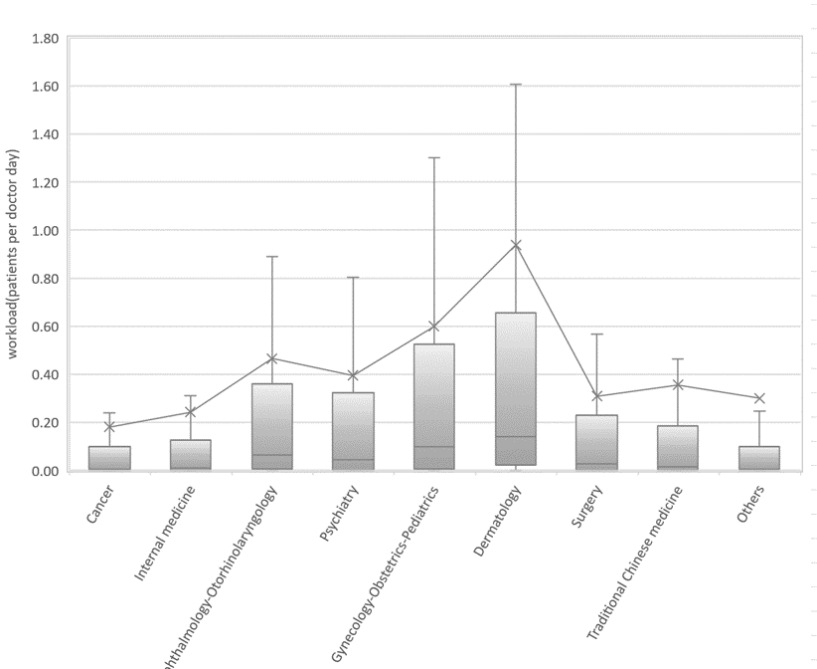
Distribution of online and offline patients per doctor across departments.

Figure 4 shows the distribution of online workload across departments. With BMCP, dermatology, ophthalmology- otorhinolaryngology, and gynecology-obstetrics-pediatrics are significantly higher than other departments. The workload in psychiatry department is not significantly different from that of most other departments. The online workloads of doctors in the departments of cancer, internal medicine, surgery, and traditional Chinese medicine are lower than the above departments. With z test, the online workload of doctors is compared with the adjusted online workload, ophthalmology-otorhinolaryngology and surgery are significantly higher than the adjusted value, whereas cancer, internal medicine, psychiatry, gynecology-obstetrics-pediatrics, dermatology, and traditional Chinese medicine are significantly lower than the adjusted value.

**Figure 5:**
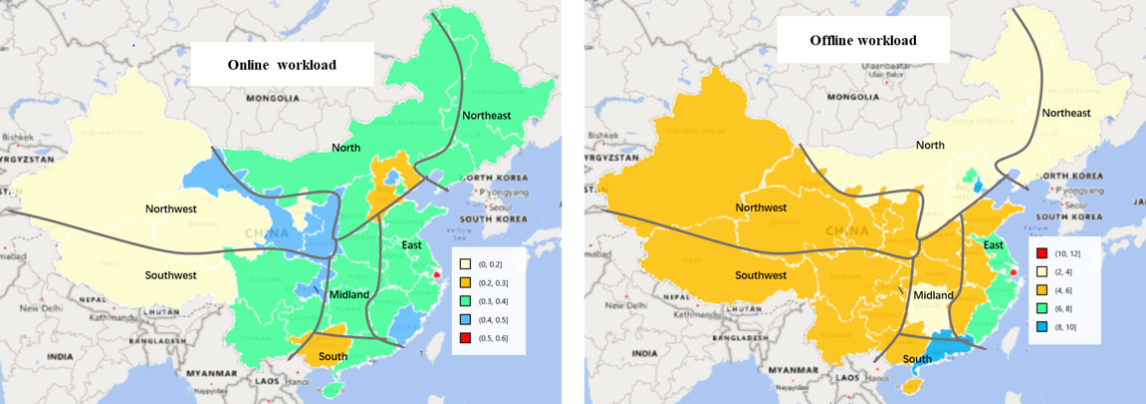
Workload for online and offline doctors in Mainland China.

Figure 5 shows the workload of online and offline doctors in the provinces of China. The offline workloads in Beijing, Shanghai, and most coastal provinces and cities are above 6, whereas the offline workloads in other provinces range from 2 to 6, with no big difference across these provinces. There are 6 provinces with online workloads lower than 0.3 located in the north and west of China. However, the online workloads for most provinces range from 0.3 to 0.4. Beside Beijing and Shanghai, there are also several provinces located in the west of China with online workloads higher than 0.4. In summary, the online workloads are more evenly distributed in cyberspace than offline.

### What Is the Price for the Services?

**Figure 6:**
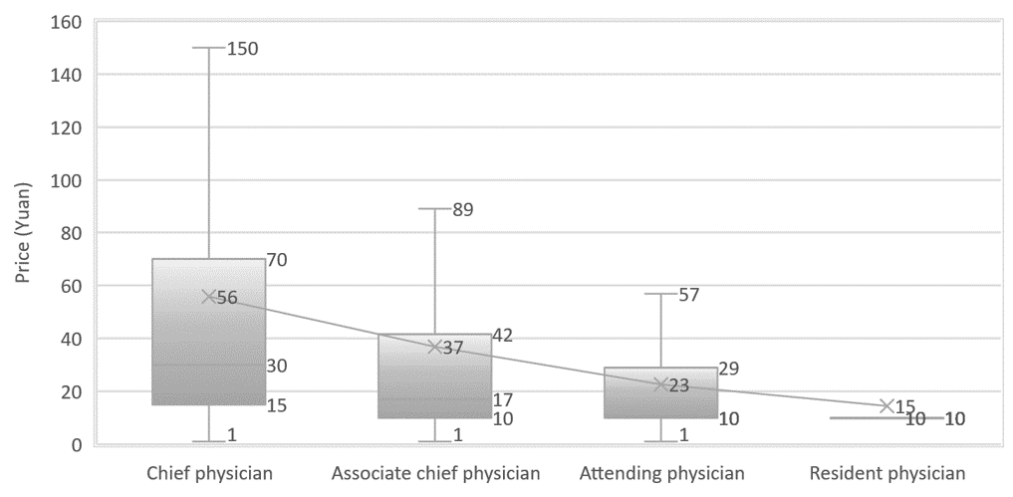
Distribution of price of Web-based consultations across various doctors’ titles.

**Figure 7:**
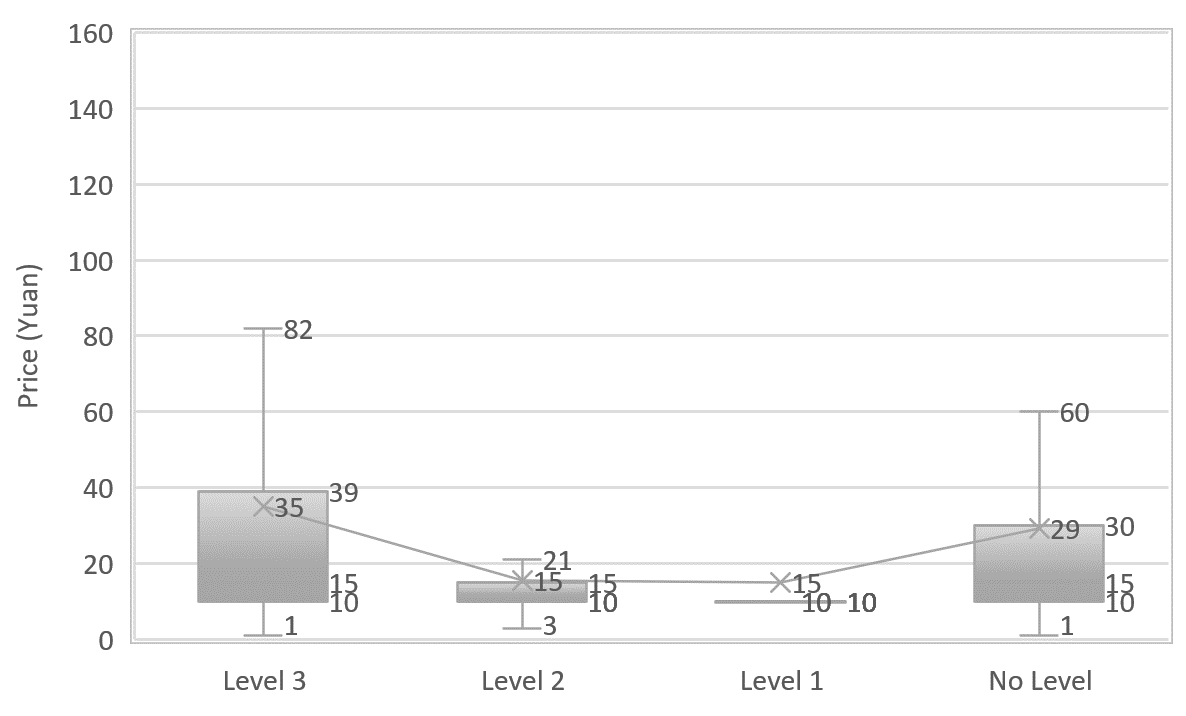
Distribution of price of Web-based consultations across hospitals.

**Figure 8:**
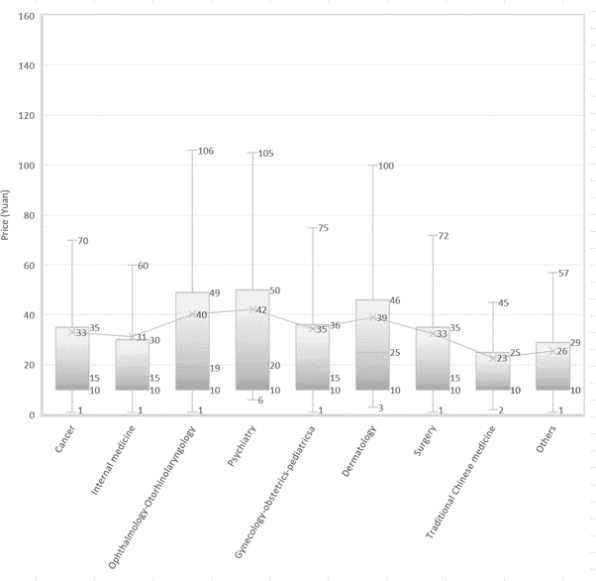
Distribution of price of Web-based consultations across departments.

We show the price distribution in Figures 6-8. The average price of a Web-based consultation is ¥32.3. More than 75% of doctors charge no more than ¥32.3—the average fee. The average fee for chief physicians (CFee, ¥56), associate cheif physicians (ACFee, ¥37), attending physicians (AFee, ¥23), and resident physicians (RFee, ¥15) decrease with the decline in experience and skill level. With BMCP, the descending order (CFee > ACFee, ACFee > AFee, AFee > RFee) are significant at .05. The average price for Web-based consultation by doctors from level 3 hospitals is ¥35, twice that of doctors from level 2 and level 1 hospitals.

The price distribution of Web-based medical consultation service varies greatly among different departments. According to the result of BMCP, doctors from psychiatry, gynecology-obstetrics-pediatrics, and dermatology charge more than doctors in other departments. Doctors from traditional Chinese medicine charge an average fee of ¥23, which is lower than any other department.

**Figure 9:**
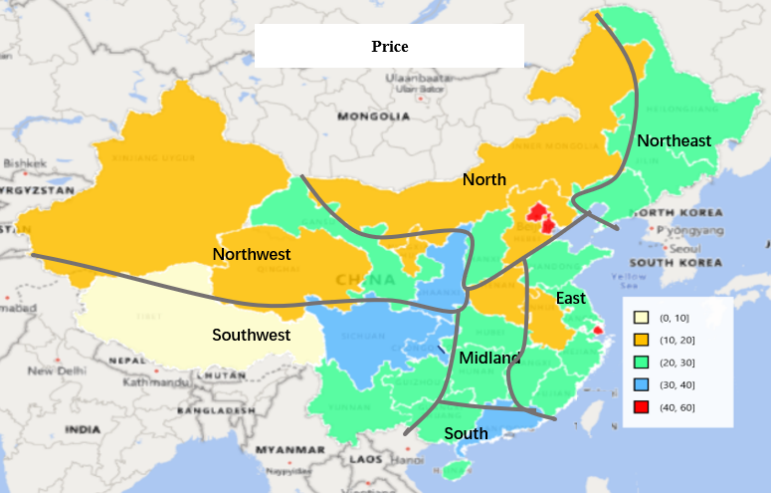
Distribution of price of Web-based consultations across provinces in Mainland China.

As shown in Figure 9, doctors from Beijing, Tianjin, and Shanghai charge the highest prices for Web-based consultation services, more than ¥40. In most provinces of eastern and central China, consultation fees range from ¥20 to ¥30. Doctors from western and northern China charge an average fee of no more than ¥20.

## Discussion

### Principal Findings

We described the status of doctors’ Web-based consultation service on Haodf in September 2017. The platform has become a market for doctors and patients to exchange Web-based consultation service. In China, 6.1% of doctors visit the Web-based consultation platform frequently [14]. There are 88,308 doctors providing Web-based consultation on Haodf in their spare time, accounting for 5.3% of all doctors in China. The adoption rate of Haodf by doctors is higher than of other Web-based consultation platforms [14]. The platform covered doctors from nearly all medical departments all over the country. Therefore, the dataset will assist us to understand the current status of doctors’ Web-based consultation in China.

Doctors with senior titles or from top-level hospitals, which are the mark of quality service in both the online and offline environment, are more likely to provide Web-based consultation services on Haodf. Doctors with junior titles or from low-level hospitals are less competitive compared with quality doctors in the Web-based consultation platform, which decreases their positivity toward participation. Furthermore, the quality doctors have higher demand on the internet [8], and the high demand attracts more quality service providers to enter the Web-based consultation market.

Quality doctors are more appealing to patients in the online environment than offline. At the same time, the fees for Web-based consultation service are highest for quality doctors with senior titles or from top-level hospitals. This shows the existence of the Matthew Effect: quality doctors who get more patients and wealth offline than other doctors could get even more resources from an online environment [8]. With patients’ increased demand for quality doctors, the platform could help quality doctors to make good use of their spare time to serve more patients. Web-based platforms reduce the cost of searching and accessing quality health care. A large number of quality doctors from all over the country serve on Web-based consultation platforms, providing patients with more options than offline environments. The platform also collects a large amount of information about doctors to provide references for patients, including reputation and experience. With this information, patients can easily find quality doctors from all over the country. Without time and space limitations, patients can communicate with any doctor on the platform through the internet.

### Price of the Web-Based Consultation Service

The average price for Web-based consultation service is ¥32, which is higher than the price of offline consultation. The doctor’s service is priced mostly by the government and the hospital, especially in the public hospitals, which account for the majority of hospitals in China. For example, the offline consultation price in Anhui is from ¥1 to ¥20 [18] and in Hubei is no more than ¥25 [19,20]. However, the price of Web-based consultation is lower than that of offline consultation in some developed countries. A traditional office visit typically costs US $150 in the United States [21,22] and £100 to £250 in the United Kingdom [23]. Teladoc (from the Unites States) charges US $49 for each consultation, whereas Mdlive (from the United States) charges US $45 per consultation and PushDoctor (from the United Kingdom) charges £20 for 1 consultation. The cost of time could be a reason for the difference. In China, an offline consultation usually takes about 2 min [24]. Offline consultation usually takes 21 min in the United States and 15 min in the United Kingdom [25]. Web-based consultation could be an unlimited asynchronous message communication or 10 to 20 min through cell phones or videos, which will cost more of the doctor’s time than serving an offline patient. In China, the price for offline service is set by the government based on the doctor’s title, hospital’s level, and location. However, the price of Web-based consultation on the platform ranges from ¥1 to ¥1000. The price of Web-based consultation is set by doctors themselves, which reflects the value of doctors’ self-evaluation.

### Web-Based Consultation Service in Each Department

There are wide variations in the status of Web-based consultation among doctors in different departments. Table 3 compares the providing of Web-based consultation in each department. In dermatology, ophthalmology- otorhinolaryngology, and gynecology-obstetrics-pediatrics, the online doctor ratio, the online doctor workload, and the price are higher than their average levels, respectively. Doctors in these departments like to provide Web-based services and sell their service at a higher price. This shows good development of a submarket in these departments. In dermatology, the visual examination of the skin is often the key part of the consultant’s physical examination [26]. The visual nature makes dermatology an obvious candidate for telemedicine techniques, and the feasibility and reliability of teledermatology is already well established [27]. The well-developed online market for gynecology-obstetrics-pediatrics should result from the high demand for quality service in this department. There is a shortage of doctors for this department in China [28], which leads to overcrowding in hospitals. The less crowded online consultation platform could be an alternative choice. Although children are always the focus of a family and the extended family in Chinese culture, an Web-based consulting service that is not limited by time or space is ideal if an urgent need arises. Web-based consultation in psychiatry has occurred since the mid-1990s [29]. There are no known absolute exclusion criteria or contraindications for any specific psychiatric diagnoses, treatments, or populations [30]. Psychiatry is an ideal fit for Web-based consultation [30]. In traditional Chinese medicine, compared with the high offline patient number per doctor, the low online doctor ratio, online workload, and price show this is a poorly developed online submarket. Traditional Chinese medicine practitioners prefer the 4 ways of diagnosis— “look, listen, question and feel the pulse” [31], which are difficult to do in the online environment. In cancer and surgery, the high online ratio and the low online workload show the excess supply of doctors and low demand in these 2 departments. High remuneration in these 2 departments leads medical students to choose these specialties [32], which decreases the patient numbers for each doctor. Providing Web-based consultations could give doctors an optimal opportunity to access more patients. Furthermore, Web-based consultations are a good opportunity to give or get a second opinion. In internal medicine, online doctor ratio, online patient number per doctor, and the price are lower than the average values. To cope with the aging population and increase in chronic diseases, the government has increased the allocation of medical resources in internal medicine, which temporarily leads to more doctors and fewer patients per doctor. The low online ratio may be because some physical examination, which is the basis for diagnosis, is not possible on the platform.

**Table 3:** Comparison of Web-based consultation provision in each department.

Department	Online ratio	Price	Online workload	Adjusted online workload
Cancer	+^a^	+	−^b^	−
Internal medicine	−	−	−	−
Ophthalmology-otorhinolaryngology	+	+	+	−
Psychiatry	+	+	N^c^	−
Gynecology-obstetrics-pediatrics	+	+	+	+
Dermatology	+	+	+	−
Surgery	+	+	−	+
Traditional Chinese medicine	−	−	−	−
Others	−	−	−	−

^a^The value is significantly higher than the average value of all online doctors.

^b^The value is significantly lower than the average value of all online doctors.

^c^The value has no significant difference from the average value of all online doctors.

### Web-Based Consultation Service in Provinces

Beijing and Shanghai have higher online ratio, workload, and fees for Web-based consultations than other provinces. As the economic centers of China, Beijing and Shanghai have more top hospitals [33] and more famous doctors [34]. The quality medical resources attract patients from all over the country. Without transportation cost, Web-based consultations become an excellent way to access the quality medical service in Beijing and Shanghai. Quality doctors and easy access lead to the high demand for Web-based service of doctors in these places. The quality and large demand result in higher fees for the Web-based service of these doctors. The high demand and high remuneration encourage the doctors’ participation, which shows as the high online ratio. Furthermore, having centralized quality doctors leads to fierce competition among doctors in Beijing and Shanghai. Reputation is a good way to stand out from the crowd, and Web-based service is a good way to build reputation. The Web-based platform can be an optional channel for doctors to communicate with offline patients as well.

The workloads are more evenly distributed across provinces in the online environment. This may be because of the platform’s triage and market power. The platform has a large number of online doctors from all over the country and shares their detailed information. The platform helps patients find the right doctor nearby rather than contending for quality doctors who are overbooked. The platform could balance the workload among all its doctors with appropriate professional competence. Compared with the offline market, which is affected by the government’s macroplan, the online market is easier to enter and leave. Doctors could become an online doctor by connecting to the internet. The internet covers 55.8% of people in China, more in cities [35], and doctors are more likely to adopt the internet. Nearly all offline doctors are potential online market entrants. Doctors could simply choose to enter or leave the market according to the market size.

It is well to remember that Ningxia Autonomous Region, located in west China where the economy is less developed and medical resources are scarce, has a higher percentage of doctors online than all other provinces except Beijing and Shanghai. This could be caused by the policies and the government’s intervention. The local government of Ningxia Autonomous Region published *Yinchuan Internet Hospital Management Working System (trial)* and *Yinchuan Internet Medical Institution Supervision and Management System (trial)* in August 2016. They are the first internet hospital regulations in China. The promotion policies are directly related with the doctor’s income and attract doctors to provide online consultation. At the same time, the online workload and service fee are very low compared with other provinces. This shows that policies could guide doctor participation although they may not attract patients.

### Conclusions

There are 88,308 doctors providing Web-based consultation in their spare time on Haodf as of September 23, 2017, accounting for 5.3% of all doctors in China. There are 0.38 patients per online doctor per day on average. The average price of Web-based consultation is ¥32.3, which is higher than the price for offline consultation. The online submarkets for dermatology, psychiatry, and gynecology-obstetrics-pediatrics are better developed than for other departments such as internal medicine and traditional Chinese medicine. Government policy could promote doctors’ participation in Web-based consultation.

The first contribution of this paper is that it is the first study, or one of the first, to examine the status of the provision of Web-based consultation in China. This study explores the status of doctors in each department and location providing Web-based consultation services. This paper could be a reference for Web-based medical service providers to establish Web-based consultation services, for doctors to make decisions about Web-based consultation, and for government policy making.

### Limitations

This research has some limitations. First, we included just one of the Web-based consultation platforms, although it is the most acceptable one. Second, we only have the aggregate data of all offline doctors in China instead of each doctor’s detailed information.

## Abbreviations

BMCP: Bonferroni multiple comparison procedure
